# Quantifying cerebral autoregulation following endovascular thrombectomy using wavelet transformation

**DOI:** 10.3389/fneur.2026.1779564

**Published:** 2026-06-16

**Authors:** Lars Tveit, Maria Skytioti, Thor Håkon Skattør, Brian Anthony Enriquez, Anne Hege Aamodt, Karolina Skagen, Markus Wiedmann, Mona Skjelland

**Affiliations:** 1 Institute of Clinical Medicine, Faculty of Medicine, University of Oslo, Oslo, Norway; 2 Department of Neurology, Oslo University Hospital, Oslo, Norway; 3 Institute of Basic Medical Sciences, Faculty of Medicine, University of Oslo, Oslo, Norway; 4 Division of Radiology and Nuclear Medicine, Oslo University Hospital, Oslo, Norway; 5 Department of Neuromedicine and Movement Science, Faculty of Medicine, Norwegian University of Science and Technology, Trondheim, Norway; 6 Institute of Population Health, Faculty of Health and Life Sciences, University of Liverpool, Liverpool, United Kingdom; 7 Department of Neurosurgery, Oslo University Hospital, Oslo, Norway

**Keywords:** cerebral autoregulation, cerebral blood flow, cerebral hemodynamics, continuous wavelet transform, neuromonitoring

## Abstract

**Introduction:**

Cerebral autoregulation (CA) is essential for protecting the brain against harmful fluctuations in cerebral blood flow (CBF) and may be severely impaired in acute ischemic stroke (AIS) patients. Continuous wavelet transform (CWT) is a robust method for quantifying CA. However, data on CWT-based metrics following endovascular thrombectomy (EVT) remain limited. Therefore, we aimed to characterize CA using CWT in AIS patients after EVT.

**Methods:**

We performed a prospective observational study recruiting patients with anterior circulation AIS treated with EVT. Continuous bilateral middle cerebral artery (MCA) blood flow velocities were obtained by transcranial doppler ultrasound (TCD) and synchronized with arterial blood pressure (ABP). CWT-derived Synchronization index gamma (SI) was calculated in the very-low frequency range (0.005–0.08 Hz). The primary outcome was an unfavorable clinical outcome at 90-days (modified Rankin Scale 3–6). Secondary outcomes were early neurological recovery, Alberta Stroke Program Early CT Score (ASPECTS), MRI-based infarct volume, and intracranial hemorrhage.

**Results:**

A total of 20 patients were included in the primary outcome analysis. There was no statistically significant difference in median Synchronization index gamma (SI) between patients with unfavorable clinical outcomes compared to patients with favorable clinical outcomes (0.61 ±0.15 vs. 0.41 ±0.20, *p* = 0.055). Patients who failed to achieve early neurological recovery had higher median SI compared to others (0.63 ±0.14 vs. 0.36 ±0.17, *p* = 0.005). Median SI was inversely correlated with ASPECTS, indicating impaired CA in larger infarcts (Spearman's rho = −0.52, *p* = 0.043).

**Conclusion:**

CWT is a viable method for evaluating CA after EVT and may provide important information on early cerebral hemodynamics following arterial recanalization.

## 1 Introduction

Timely vessel recanalization by intravenous thrombolysis and endovascular thrombectomy (EVT) is the hallmark of acute ischemic stroke (AIS) treatment (1). Despite significant advances in therapeutic interventions and delivery of care, AIS still carries a substantial risk of severe disability and death (2, 3). Even after successful arterial recanalization, unfavorable outcomes with physical and cognitive impairments are common (1, 4, 5). There are multiple causes contributing to this discrepancy, collectively referred to as futile recanalization. This may involve impaired microvascular reperfusion (3, 6), irreversible ischemic damage prior to treatment (3) and reperfusion injury (7–10) among other contributing factors. Furthermore, the regulatory mechanisms controlling cerebral blood flow (CBF) are frequently impaired in AIS, which may lead to dysregulation of cerebral perfusion (11). AIS patients may be particularly vulnerable to disturbances in CBF, as hypoperfusion can result in accelerated deterioration of penumbral volume (12), while hyperperfusion, combined with blood–brain barrier disruption, may increase the risk of bleeding (13, 14).

*Cerebral autoregulation* (CA) is essential for securing stable CBF despite variations in cerebral perfusion pressure (CPP) (15, 16). It is one of several partly overlapping systems modulating brain perfusion (15, 17) and plays a critical role in protecting the brain against injury due to hypo- and hyperperfusion (16, 18). Dynamic cerebral autoregulation describes the cerebrovascular response to swift and temporary alterations in arterial blood pressure (ABP) over short durations (15, 19). ABP is constantly varying due to everyday activities, as well as at rest due to breathing and spontaneous fluctuations (20). These variations have an oscillatory nature and can be categorized by frequency. CA is often described as having the properties of a *high-pass filter*, where fluctuations in ABP occurring at higher frequencies (>0.08 Hz) are readily transmitted to the cerebral circulation, while low-frequency oscillations are more effectively buffered (15).

There are multiple approaches, both in estimating CBF and in calculating CA metrics (19, 21). CBF velocities can be assessed using transcranial doppler ultrasound (TCD) (22), offering safe and non-invasive measurements with high temporal resolution in various clinical settings. Impaired CA is frequent in stroke patients (16, 23, 24) and has been linked to poor functional outcome and increased infarct size (11, 25). Furthermore, the detection of early CA impairment has been associated with hemorrhagic transformation (18) and cerebral edema (26). CA estimation using *continuous wavelet transform* (CWT) is a robust alternative to methods such as transfer function analysis (TFA) or correlation-based methods (19, 27–30). CWT has previously been applied in several clinical settings (28, 31, 32). However, data on CWT-based metrics in the post-recanalization phase remains scarce.

This study aimed to assess early changes in CA following EVT for acute stroke. Utilizing TCD-based CBF velocities, we characterized CA using CWT in anterior circulation AIS and compared estimates to patient-related outcomes.

## 2 Materials and methods

### 2.1 Study design, setting, and participants

In this prospective observational study, we recruited patients with anterior circulation AIS treated with EVT at Oslo University Hospital (a tertiary stroke center) between May 2023 and December 2024. The eligibility criteria were AIS treated with EVT due to occlusions of the terminal internal carotid artery (ICA), main stem (M1), or first branches (M2) of the middle cerebral artery (MCA) on presenting CT angiography. Exclusion criteria were a lack of MCA doppler signal ipsilateral to the occlusion and TCD examination performed >72 h from recanalization. Written informed consent was obtained from all patients or patients' next-of-kin. The study was approved by the Regional Research Ethics Committee (REK sør-øst C, reference numbers 373176 and 13400) and the Oslo University Hospital Data Protection Officer.

### 2.2 Examination protocol

Bilateral MCA insonation was performed using 2 MHz ultrasound probes (WAKIe, Atys medical, France) stabilized with a headframe fixation device. The MCAs were identified based on probe angulation, blood flow direction, blood flow velocities, and vessel depth. Prior to data collection, sufficient signal-to-noise ratio and correct envelope tracking were ensured. Representative examples of MCA waveforms of satisfactory and unsatisfactory signal quality are provided (Supplementary material S1). All TCD examinations were performed by a neurologist experienced in neurosonology (LT). Continuous invasive ABP measurements (radial artery catheter) were synchronized with CBF maximum velocities and exported for analysis at 100 Hz. Recordings were inspected to ensure beat-to-beat synchronization and verify waveform quality. Only samples of adequate quality and a duration of at least 15 min were used for further analysis.

### 2.3 Clinical and imaging variables

Patient data on demographics, comorbidities, and medication use were systematically recorded. Clinical data pertaining to the stroke event, including presenting National Institutes of Health Stroke Scale (NIHSS), use of thrombolytics, and use of general anesthesia, were registered. Imaging parameters including thrombus occlusion site, Alberta Stroke Program Early CT Score (ASPECTS) at baseline and 24 h after EVT (based on CT or MRI), infarct volume (based on diffusion weighted imaging), Thrombolysis in Cerebral Infarction (TICI) score, and intracranial hemorrhage as judged by the Heidelberg bleeding classification (HBC) (33) were assessed by an interventional radiologist (THS) blinded to CA metrics. To limit the impact of small, punctate lesions on infarct size in diffusion-weighted imaging (DWI)-ASPECTS, a region was classified as affected only if it contained an ischemic lesion with a largest diameter of at least 10 mm (34). ASPECTS was analyzed both as an ordinal outcome variable as well as dichotomized into small and large infarcts (ASPECTS 0–5). Clinical outcome was assessed by the modified Rankin Scale (mRS) at 3 months, obtained through telephone interview or by clinical control by a physician blinded to CA metrics. mRS was dichotomized into favorable (mRS 0–2) and unfavorable outcomes (mRS 3–6). Early neurological recovery was defined as NIHSS ≤ 8 at 24 h (35). Symptomatic intracranial hemorrhage (sICH) was defined as any intracranial hemorrhage combined with an increase in NIHSS of ≥4 points.

### 2.4 Continuous wavelet transform analysis

To quantify CA, CWT was applied by analyzing the relationship between spontaneous oscillations in ABP and bilateral MCA blood flow velocities (36, 37). Two CA metrics were calculated: the phase synchronization index gamma (SI) (37) and the wavelet-based phase coherence (WPC) (38) within the very low frequency (VLF) range (0.005–0.08 Hz). The SI shows the phase difference variability, with low values indicating intact CA (32, 37, 39). WPC describes the phase relationship between two fluctuating variables at a specific frequency; similarly to SI, low values of WPC indicate preserved CA. Although not the same metric, both SI and WPC range from 0 to 1 and are known to be strongly correlated (40). The peak (maximum) and the median SI and WPC over the VLF range were calculated for both hemispheres (36, 41). The time-frequency analysis tools employed in the present study have been developed by the Department of Physics, Lancaster University, UK (38, 42–44).

### 2.5 Statistical methods

Numerical variables were summarized as medians with interquartile ranges (IQR) or means with standard deviations (SD), depending on their distribution. Categorical variables were summarized as frequencies with percentages. The primary exposure variable was the median SI in the VLF range (0.005–0.08 Hz) calculated from the MCA signal ipsilateral to vessel occlusion. The primary outcome was an unfavorable clinical outcome at 90-days (mRS 3–6). Secondary outcomes were early neurological recovery, ASPECTS, infarct volume, and intracranial hemorrhage. Between-group comparisons for early neurologic recovery, clinical outcome, and intracranial hemorrhage were performed using the Mann–Whitney *U* test. ASPECTS and infarct volume were correlated with median SI using Spearman's rank correlation. Additional analyses included Pearson correlations between median/peak WPC and SI and ranked differences in median SI between subgroups (comorbidities, TICI score). To account for multiple testing, *p*-values presented for primary and secondary outcomes have been adjusted using the Benjamini–Hochberg procedure to control the false discovery rate. Analyses were performed on available cases, and no imputation was applied for missing data. *p*-Values < 0.05 were considered statistically significant. Statistical analyses were performed using STATA version 19 (StataCorp LCC, TX, USA).

## 3 Results

**Figure 1:**
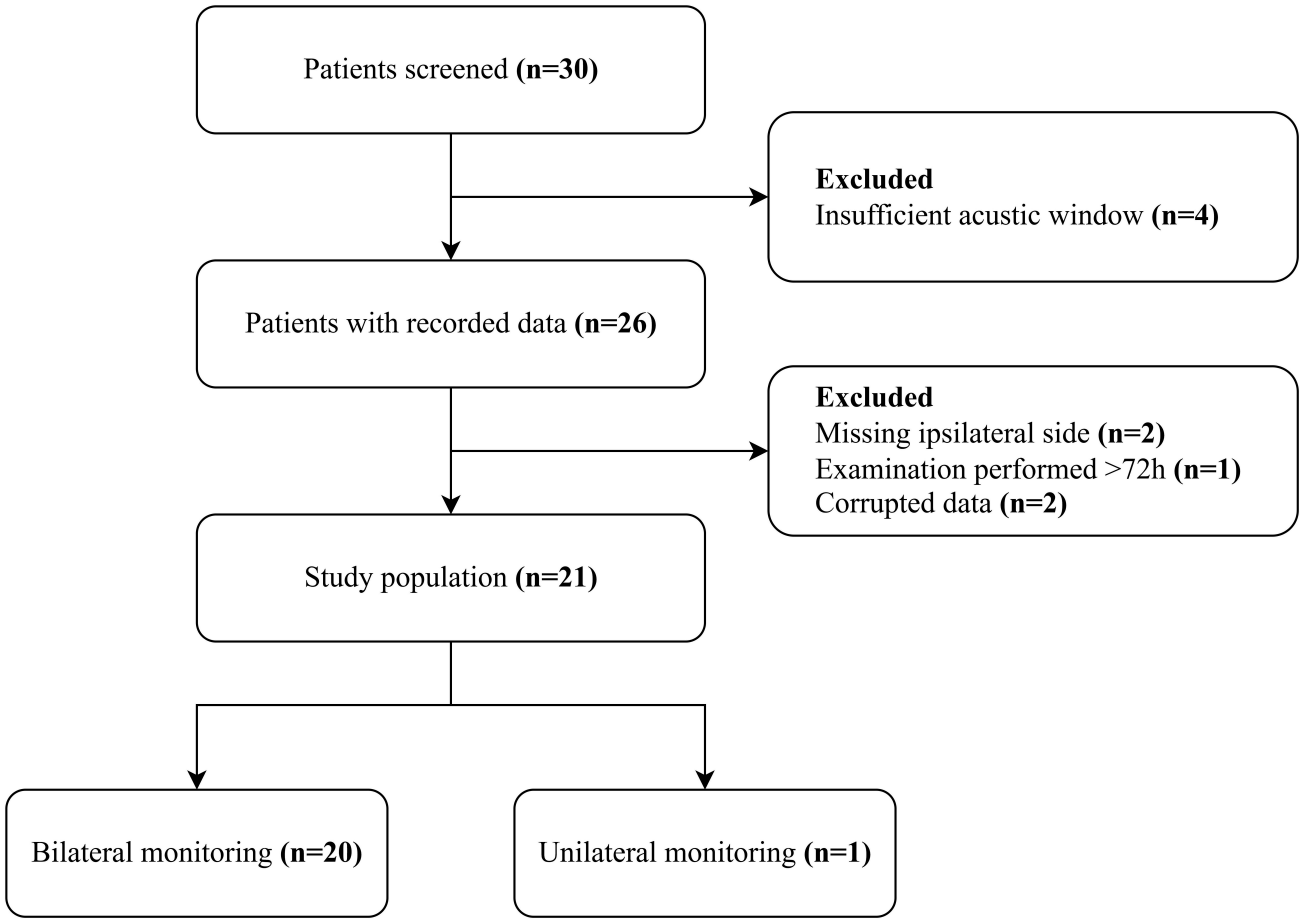
Flow diagram detailing number of patients screened, reasons for exclusion and final study population.

**Figure 2:**
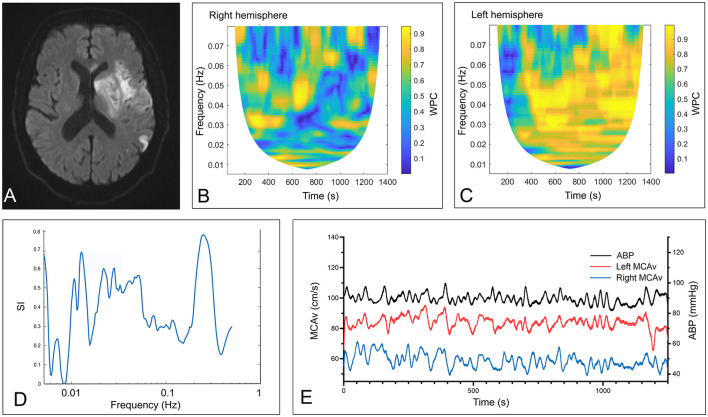
Example case. A 76-year-old male patient with known hypertension experienced sudden onset of right-sided hemiplegia and aphasia. NIHSS was 19 at hospital admission, and CT angiography revealed left-sided M1 occlusion. He was treated with intravenous thrombolysis and EVT, achieving TICI 2B; 90 days mRS was 4. **(A)** Brain MRI 37 h post-recanalization displaying diffusion restriction in the left MCA territory. ASPECTS and MRI-based infarct volume were estimated to 3/10 and 96 ml, respectively. **(B)** Contour plot displaying WPC (color intensity) in the non-ischemic hemisphere. Warm colors denote increased WPC, indicating impaired CA. Frequency (Hz) in the very low frequency range is plotted against the duration of recording (s). **(C)** Contour plot of hemisphere ipsilateral to vessel occlusion. Note increased WPC over a broad frequency range. **(D)** SI in the ipsilateral hemisphere plotted against frequency (Hz, logarithmic scale). Note SI peak at 0.013 Hz, indicating pronounced cerebral dysregulation within the autoregulatory range. **(E)** Synchronized recording of bilateral MCA blood flow velocities shown together with invasive ABP displaying spontaneous oscillations. ABP Arterial blood pressure; ASPECTS Alberta Stroke Program Early CT Score; EVT indicating endovascular thrombectomy; mRS MCA Middle cerebral artery; modified Rankin Scale; NIHSS National Institutes of Health Stroke Scale; SI Synchronization index; TCD Transcranial doppler ultrasound; TICI Thrombolysis in Cerebral Infarction; WPC Wavelet-based phase coherence.

Among 30 patients eligible for study inclusion, nine patients were excluded, resulting in a final sample of 21 patients included in the analysis (Figure 1). Of these, one patient was lost to 90-day follow-up. The median age was 71 years (IQR 59–76), and 14 were male (67%). Patient characteristics are summarized in Table 1. All except one patient had bilateral TCD monitoring. Figure 2 illustrates autoregulatory analyses from a representative patient. TCD was performed at a mean of 27.6 h (SD ±12.7) after recanalization. All patients underwent brain imaging after EVT, and postinterventional MRI scans were obtained in 17 (81%) of them. Vasopressors (noradrenaline) were administered in two patients (10%) during monitoring. Clinical data and contour plots of all CA analyses are available in the Supplementary material S1.

**Table 1:** Patient characteristics (*n* = 21).

Variables	Values
Age, median (IQR)	71 (59–76)
Female sex, *n* (%)	7 (33%)
mRS av admission, median (IQR)	0 (0–0)
Comorbidities, *n* (%)	
Diabetes	2 (11%)
Hypertension	9 (50%)
Atrial fibrillation	2 (12%)
Smoking, *n* (%)	4 (31%)
Mediation use	
Platelet inhibitors	3 (17%)
Anticoagulation	1 (6%)
Antihypertensives	9 (50%)
Statins	6 (33%)
Occluded vessel, *n* (%)	
TICA	3 (14%)
M1	14 (67%)
M2	4 (19%)
Symptomatic side, *n* (%)	
Left	11 (52%)
NIHSS at admission, median (IQR)	15 (9–20)
Pre-treatment ASPECTS, median (IQR)	8 (7–9)
Use of thrombolytics, *n* (%)	14 (74%)
Use of general anesthesia, *n* (%)	18 (95%)
Carotid stenting, *n* (%)	5 (26%)
TICI score, *n* (%)	
2C/3	15 (71%)
2B	6 (29%)

mRS, modified Rankin Scale; tICA, terminal internal carotid artery; M1, first branch of middle cerebral artery; M2, second branch of middle cerebral artery; LSW, last seen well; NIHSS, National Institutes of Health Stroke Scale; ASPECTS, Alberta Stroke program early CT score; TICI, Thrombolysis in Cerebral Infarction.

### 3.1 Cerebral autoregulation metrics

**Figure 3:**
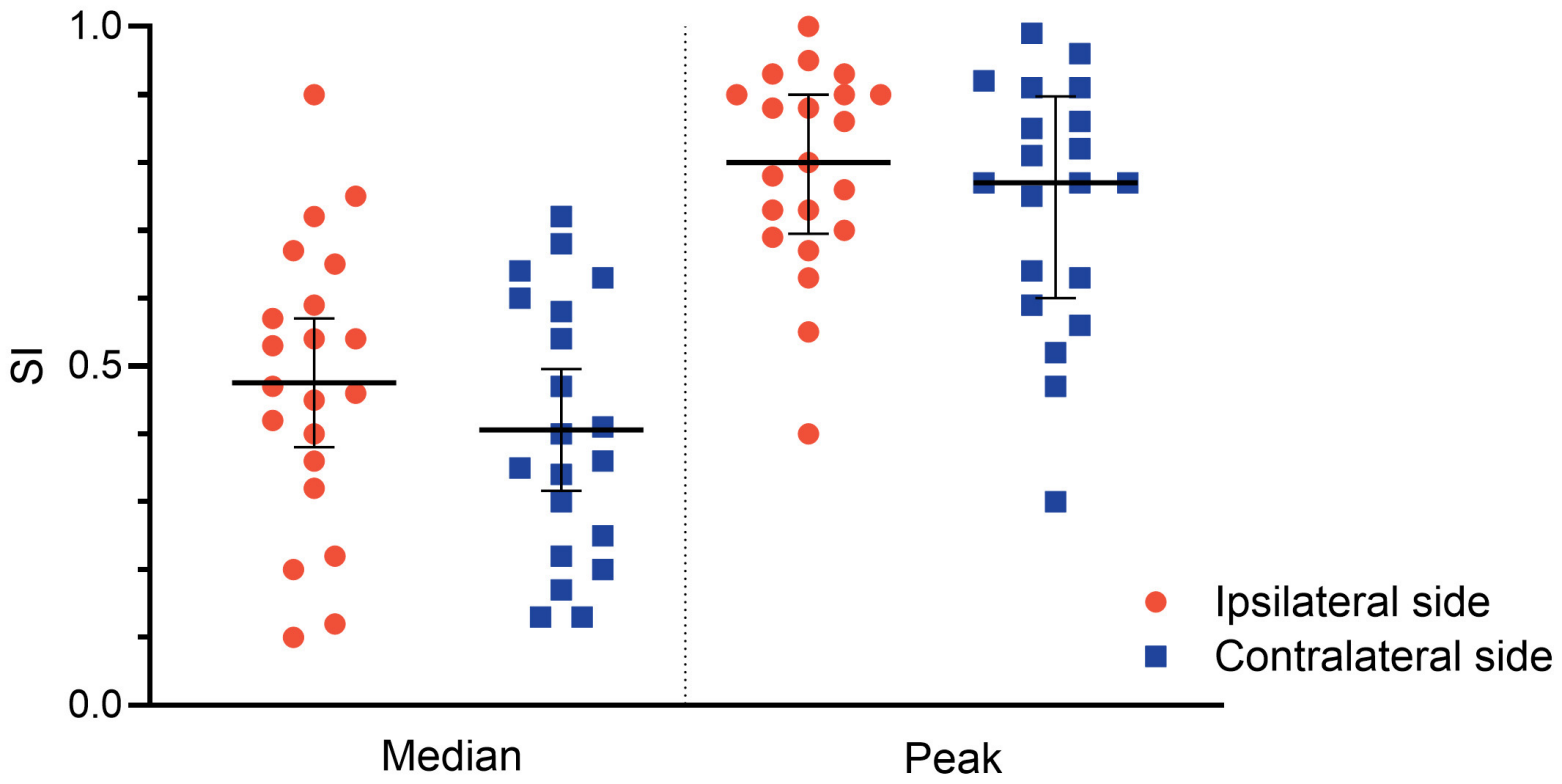
Dot plot displaying median synchronization index gamma (SI) (mean, 95% CI) and peak SI (median, IQR) of patients. CI, confidence interval; IQR, interquartile range; SI, synchronization index.

There was a linear correlation between SI and WPC when comparing both peak and median values in both hemispheres (*r* = 0.99 *p* < 0.001). Given this strong correlation, only SI is presented in the following text. Autoregulatory indices are summarized in Table 2. Median and peak SI for both hemispheres are presented in Figure 3. Patients with comorbid hypertension had higher median SI in the ipsilateral hemisphere compared patients without a history of hypertension (0.58 ± 0.16 vs. 0.36 ± 0.19, *p* = 0.013). Individual patient data including autoregulatory indices and outcomes are displayed in Table 3.

**Table 2:** Autoregulatory indices ipsilateral and contralateral to vessel occlusion.

Autoregulatory indices	Hemisphere	
	Ipsilateral	Contralateral
Wavelet phase coherence		
Median	0.68 (SD ±0.16)	0.62 (SD ±0.16)
Peak	0.92 (IQR 0.84–0.95)	0.88 (IQR 0.76–0.95)
Synchronization index gamma		
Median	0.49 (SD ±0.20)	0.41 (SD ±0.19)
Peak	0.83 (IQR 0.72–0.90)	0.77 (IQR 0.61–0.89)

Values are presented as mean (±SD) or median (IQR) based on distribution. Only patients with bilateral measurements. n = 20.

**Table 3:** Individual patient data presenting occlusion site, autoregulatory indices, clinical and imaging outcomes.

Age	Sex	Occlusion	Ipsilateral SI		Contralateral SI		24h NIHSS	ASPECTS	Infarct volume	mRS	Hemorrhage
			Median	Peak	Median	Peak					
80	Male	M1	0.90	1	0.68	0.99	17	3	90	6	PH1
85	Female	M1	0.75	0.95	0.64	0.96	9	6	21	2	HI2
71	Male	M1	0.72	0.93	0.60	0.92	16	4	46	4	PH1
58	Male	tICA	0.67	0.90	0.72	0.86	0	9	2	0	None
76	Male	M1	0.65	0.93	0.58	0.91	28	4		6	SAH
76	Male	tICA	0.59	0.90	0.25	0.47	24	0		6	None
67	Male	M2	0.57	0.73	0.34	0.81	0	10	0	0	None
72	Female	M1	0.54	0.86	0.54	0.82	16	4	51	2	None
76	Male	M1	0.54	0.80	0.13	0.30	22	3	96	4	PH1
75	Male	M2	0.53	0.88	0.47	0.85	11	6		3	SAH
62	Male	tICA	0.47	0.76	0.30	0.77	22	1	209	6	HI1
77	Male	M1	0.46	0.73	0.63	0.91	8	5	76	3	HI2
77	Female	M1	0.45	0.70	0.41	0.77	6	7	26	2	HI1
59	Male	M2	0.42	0.88	0.40	0.77	3	7	19	1	None
56	Male	M1	0.40	0.90	0.36	0.75	3	7	24	0	HI1
58	Female	M1	0.36	0.78	0.35	0.56	1	6	11	0	HI2
55	Male	M2	0.32	0.67	0.22	0.59	3	6	71	2	HI1
65	Male	ICA+M1	0.22	0.63	0.20	0.64	5	7	17	2	PH1
57	Female	M1	0.20	0.55			2	9	6		None
59	Female	M1	0.12	0.40	0.13	0.52	0	8	8	0	None
72	Female	M1	0.10	0.69	0.17	0.63	0	10		2	None

Sorted by median synchronization index gamma (SI).

WP, wavelet-based phase coherence; NIHSS, National Institutes of Health Stroke Scale; ASPECTS, Alberta Stroke program early CT score; mRS, modified Rankin Scale; HI, Hemorrhagic infarction; PH, Parenchymal hematoma; SAH, subarachnoidal hemorrhage; tICA, terminal internal carotid artery.

### 3.2 Clinical and radiologic outcomes

Median 90-day mRS was 2 and 8/20 (40%) had an unfavorable clinical outcome. There was no statistically significant difference in median SI between patients with unfavorable clinical outcomes (mRS 3–6) compared to patients with favorable clinical outcomes (0.61 ± 0.15 vs. 0.41 ± 0.20, *p* = 0.055).

**Figure 4:**
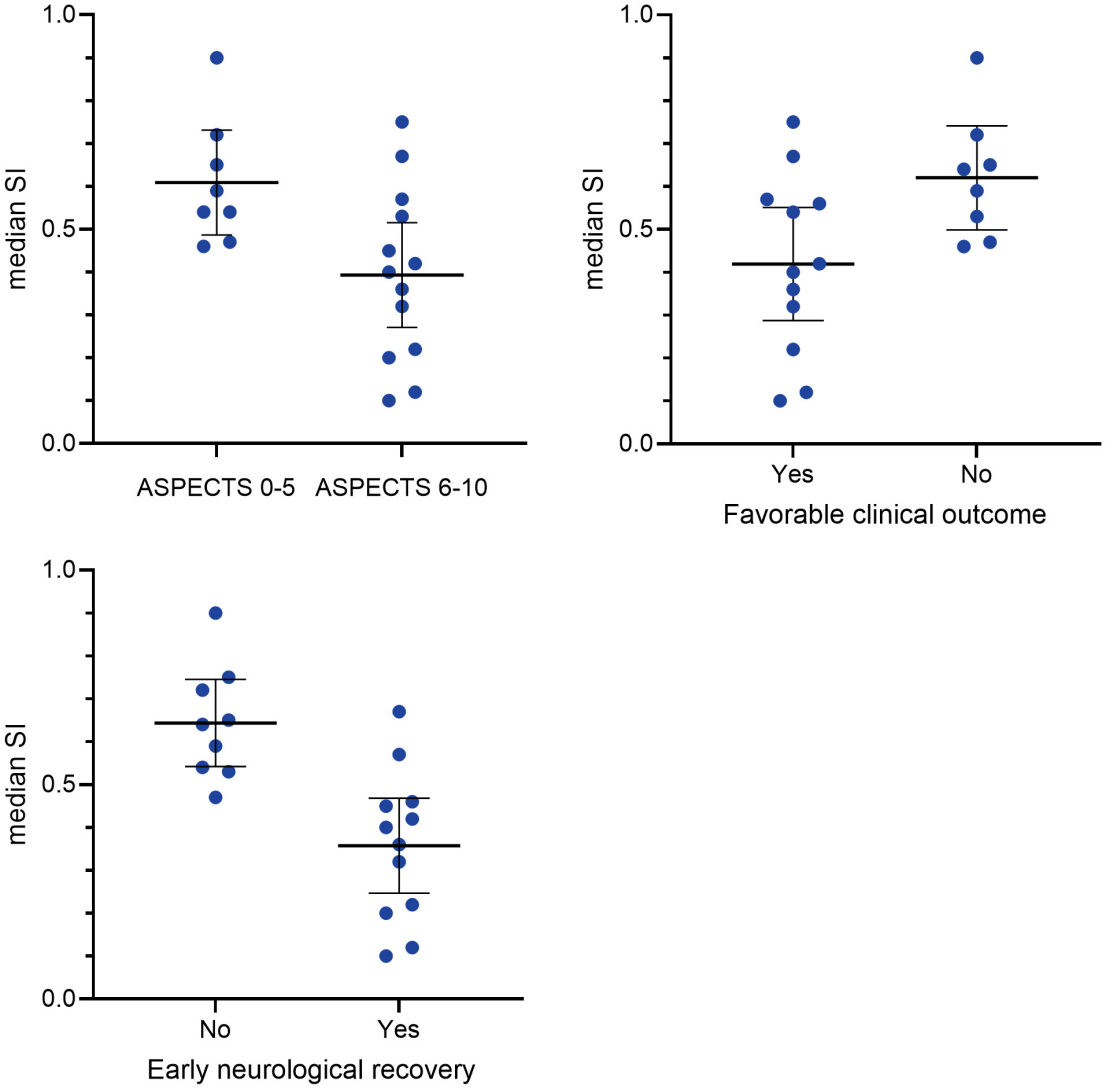
Dot plot comparing median synchronization index gamma (SI) to Alberta Stroke Program Early CT Score (ASPECTS) at 24 h, clinical outcome, and early neurological recovery. Means with 95% CI are displayed. CI, confidence interval.

Median SI was higher in patients who failed to achieve early neurological recovery compared to those who demonstrated neurological recovery, 0.63 ± 0.14 vs. 0.36 ± 0.17, *p* = 0.005. Median MRI based infarct volume was 24 ml (IQR 11–71) and median post-interventional ASPECTS was 6 (IQR 4–7). Median SI was inversely correlated with ASPECTS (Spearman's rho = −0.52, *p* = 0.043), indicating impaired CA in larger infarcts. No correlation was found between median SI and MRI based infarct volumes (Spearman's rho = 0.29 *p* = 0.257). Hemorrhage on follow-up imaging was seen in 13 patients (62%), including parenchymal hematomas (HBC type PH1) in four patients (19%). No patients had sICH. There was no difference in median SI in patients with intracerebral hemorrhage compared to those without hemorrhage. Comparisons between median SI, imaging and clinical outcomes are presented in Figure 4.

In sensitivity analyses excluding the two patients who received vasopressor treatment, median SI was inversely correlated with ASPECTS (Spearman's rho = −0.51, unadjusted *p* = 0.028) and patients with early neurological recovery had lower median SI compared to others (0.64 ± 0.14 vs. 0.36 ± 0.18, unadjusted *p* = 0.002). No difference in median SI was observed between clinical outcome groups.

All patients achieved successful recanalization (TICI ≥ 2B), and no differences were seen in median SI within this range.

## 4 Discussion

In this study we observed considerable variations in CWT-based CA metrics in the early phase following EVT, indicating pronounced differences in cerebral blood flow regulation after thrombectomy. Lower rates of early neurological recovery and higher ASPECTS were observed in patients with impaired CA, suggesting a link between CWT-derived metrics and cerebral injury post-EVT.

The cerebral dysregulation observed in our cohort may rely on a preexisting CA derangement in addition to a sudden functional compromise induced by the stroke event. Several factors influence the severity and degree of laterality of cerebral dysregulation, and it is challenging to delineate the interwoven contributions of stroke subtype, extent of ischemic damage, and comorbidities on the CA results observed in our study (15). Effective CA may impact clinical outcomes by protecting against reperfusion injury and limiting the progression of ischemic damage (11, 45, 46). Penumbral CBF prior to recanalization is a major determinant of the infarct growth rate (12) and reduced CA may escalate this progression (47). The irreversible tissue damage of an established infarct may also contribute to a volume-dependent functional disruption influencing CA metrics. Conversely, severe dysregulation despite low infarct volumes, as seen in a minority of our patients, may point to differences in patient vulnerability and non-ischemic contributors to CA impairment. As such, patients with comorbid hypertension showed impaired CA metrics compared to patients without a history of hypertension, possibly due to pre-existing cerebral endothelial and smooth muscle dysfunction (16).

A challenge in characterizing CA is the varying degree of dysregulation, even over short time periods (27, 30, 48). Furthermore, the relationship between ABP and CBF may exhibit non-linear properties (49). Addressing these characteristics, CWT-based metrics such as SI can describe variations in signal synchronization with considerable temporal resolution (27, 28, 30). In addition, the model makes no assumption of system linearity (50). These attributes highlight the advantages of CWT as an analytical method for continuous evaluation of cerebral autoregulatory status. Furthermore, the method is based on analyzing spontaneous oscillations in ABP and CBF velocities, eliminating the need for induced ABP fluctuations in a fragile population (20).

The observed SI in our report is comparable to other cohorts, including a study evaluating wavelet-based metrics in patients with minor stroke or transient ischemic attack (28). Although no matched cohort was used in the present study, we observed considerably higher SI values compared to smaller samples of healthy volunteers, suggesting impaired CA to be frequent among our patients (36, 37, 51). Our results align with previous studies indicating that impaired CA in AIS is a common finding, although methodological differences hinder direct comparison (45, 52, 53). Previous studies have reported associations between effective CA and favorable clinical outcomes (45, 53), including a meta-analysis involving 384 AIS patients (11). There may also be temporal aspects of CA dysfunction, with early normalization being related to improved outcomes (47) and poor outcomes in patients with prolonged impairment (54, 55). Risk of reperfusion injury is influenced by blood-brain barrier permeability, which shows great variability in the subacute phase of stroke (56). This, in addition to the multitude of factors modulating the risk of hemorrhagic transformation (57, 58) may partly explain the discrepant findings regarding hemorrhagic complications compared to other reports (18, 26, 53–55).

We observed a correlation between median SI and infarct size as judged by ASPECTS, suggesting impaired CA in larger infarcts. Although rank-based analysis indicated a positive correlation between median SI and MRI-based infarct volumes, no statistically significant association was observed. Infarct volume estimates displayed considerable variability and were assessed in a subset of patients, resulting in a smaller sample for analysis. Previous reports have suggested impaired CA in patients with larger infarcts, although no unified method for classifying infarct size was employed in these studies (18, 47, 59).

**Figure 5:**
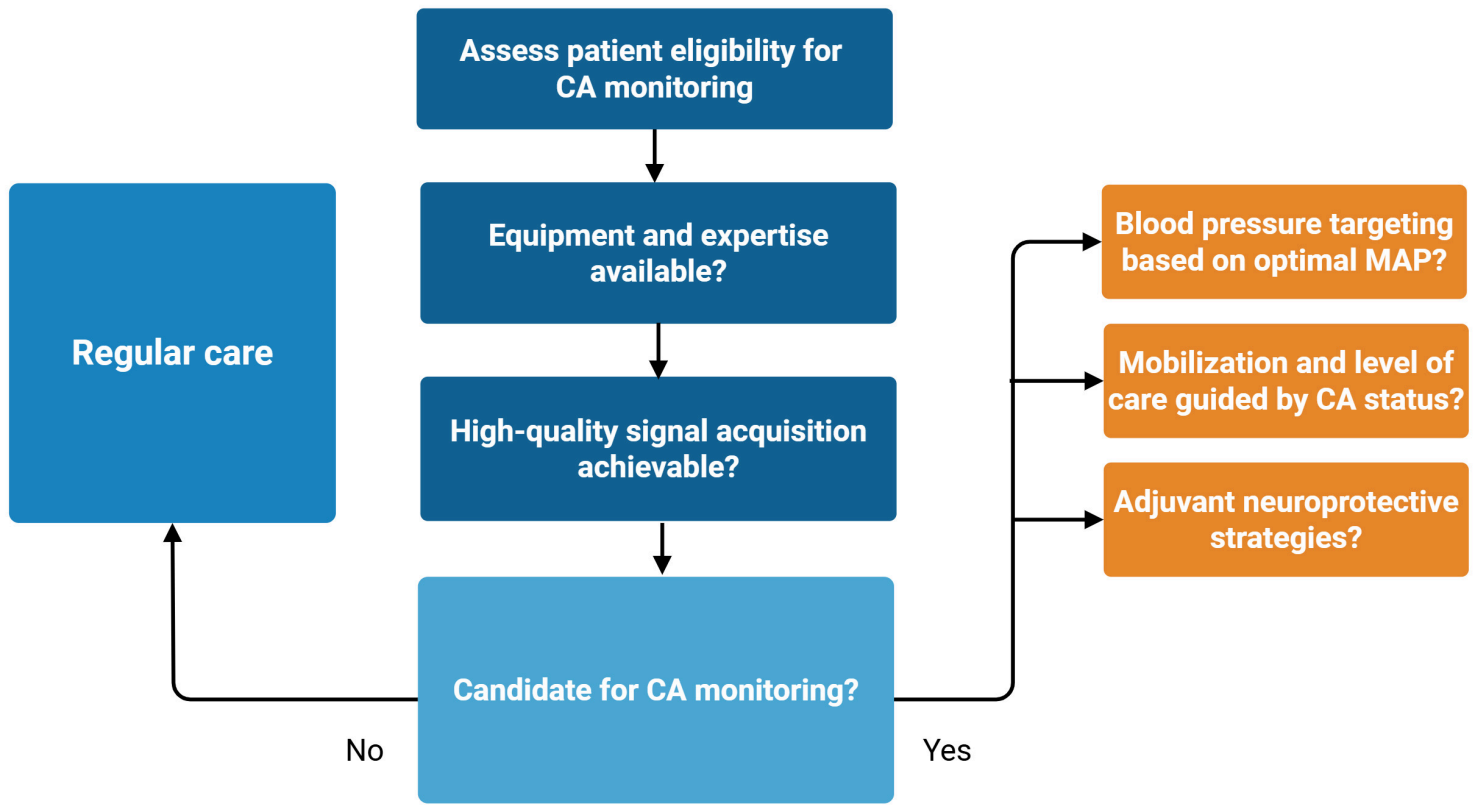
Conceptual flow chart of patient selection and potential management strategies according to cerebral autoregulation monitoring status. This schematic is intended for illustrative purposes to outline possible directions for future research. Interventional trials are required before any recommendation for routine clinical implementation can be made. Figure created with BioRender.com. CA, cerebral autoregulation; MAP, mean arterial pressure.

Impaired regulation of brain perfusion following AIS may reflect an increased risk of ischemic progression, edema formation, and hemorrhagic complications. The lack of implementation of methods evaluating these factors may lead to a uniform rather than individualized approach in patient management. Improved techniques for characterizing the degree and type of cerebral dysregulation may impact strategies for hemodynamic augmentation, neuroprotection, and level of care (Figure 5). Moreover, CA monitoring may have further potential when utilized as part of multimodal monitoring. However, it remains uncertain whether interventions based on CA metrics lead to improved outcomes, and interventional trials are needed prior to routine implementation. In addition, continuous CA quantification may be limited by the use of specialized equipment, trained personnel, potential monitoring side-effects as well as the need for post-processing of data (29).

Key limitations of this study include the small sample size, limiting statistical power, and increasing the risk of type II error. *Post hoc* power calculations indicated an estimated power of 0.65. We did not account for variations in end-tidal CO_2_, which influences cerebrovascular resistance and may affect CA estimates (17). Although imaging outcomes were assessed by a single experienced rater, independent evaluation by multiple observers would have been preferable to reduce interrater variability. In addition, TCD has a reduced spatial resolution, potentially missing smaller areas of focal dysregulation (59, 60). Strengths include TCD examinations by a single observer and outcome assessment blinded for CA data.

Despite increased insight into the regulatory mechanisms governing CBF, our understanding of CA in AIS remains incomplete. Methodological research aiming to improve usability and facilitate continuous monitoring is needed. In addition, multicenter studies should validate CWT-derived metrics and evaluate their role in neurocritical care monitoring.

## 5 Conclusion

CWT is a viable method for evaluating CA and may provide important information on early cerebral hemodynamics in the post-recanalization phase. The observed variability in CWT-derived CA metrics indicates considerable heterogeneity in cerebrovascular regulatory mechanisms following EVT.

## Data Availability

The data supporting the conclusions of this article are available from the corresponding author upon reasonable request.
