# Supplementary material for: Quantifying cerebral autoregulation following endovascular thrombectomy using wavelet transformation
Source: Front Neurol. 2026 Jun 16;17:1779564. doi: 10.3389/fneur.2026.1779564 (PMC13314523; doi:10.3389/fneur.2026.1779564)
Supplement: Supplementary file 1 [file Supplementary_File_1.pdf]

Supplementary material

Transcranial doppler ultrasound waveform examples

Middle cerebral artery (MCA) waveforms (unilateral recordings) displaying signal-to-noise ratio and envelope tracking adequate for signal processing:

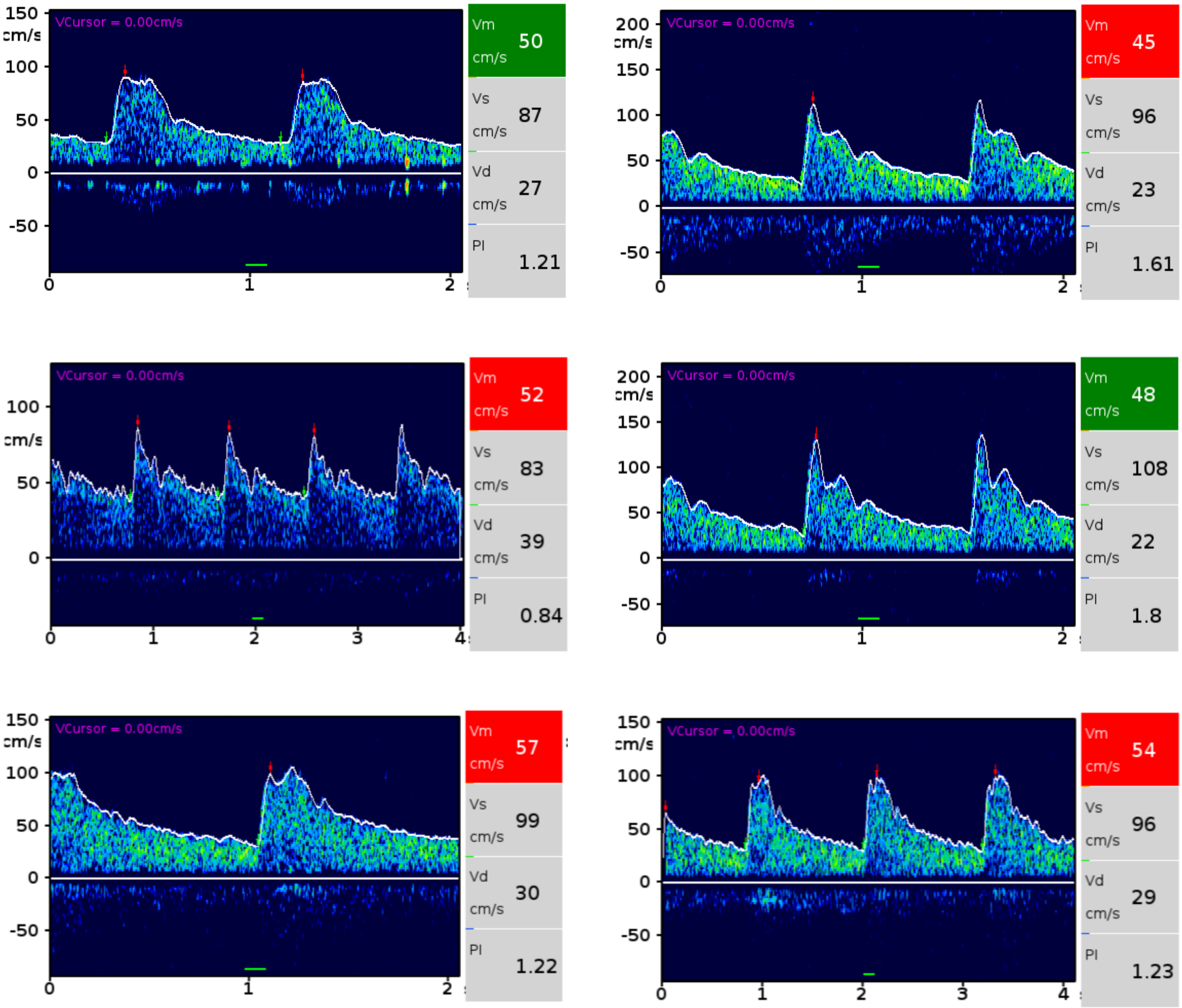

Waveform examples with low signal-to-noise ratio, incorrect envelope tracking or insonation of non-MCA mainstem target:

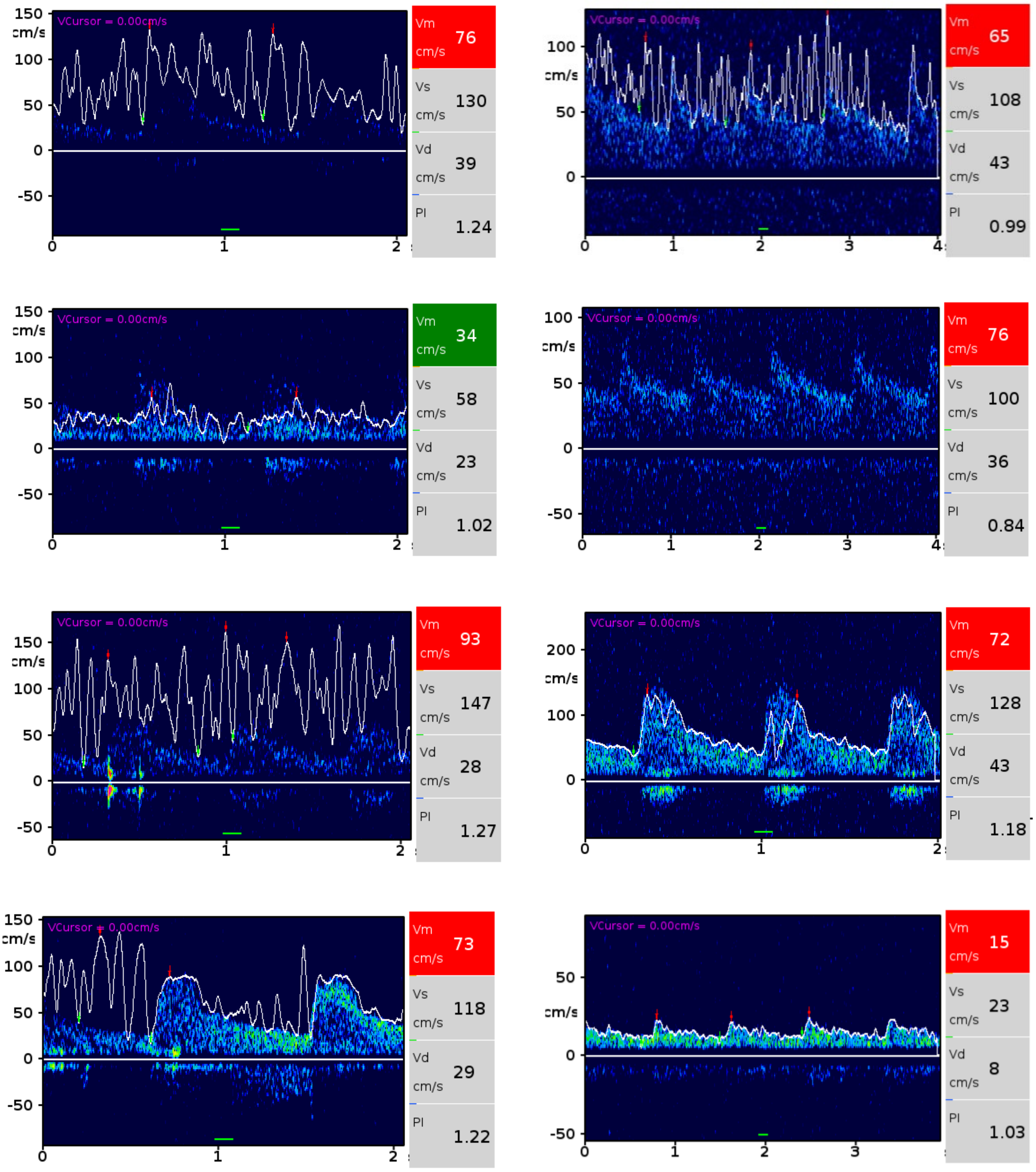

Contour plots of individual patient measurements

Contour plots displaying frequency (Hz) in the 0.005-0.08Hz range plotted against recording time (s). The color scale represents WPC, where warm colors indicate higher WPC (signaling reduced cerebral autoregulatory function). SI values are supplied for measurements from the hemisphere ipsilateral and contralateral to vessel occlusion.

NIHSS indicates National Institutes of Health Stroke Scale; mRS modified Rankin Scale; ASPECTS Alberta Stroke Program Early CT Score; WPC Wavelet-based phase coherence; SI Synchronization index

Age	Sex	Occlusion	Ipsilateral Median SI	Ipsilateral Peak SI	Contralateral Median SI	Contralateral Peak SI
59	Male	Right M2	0.42	0.88	0.40	0.77

24h NIHSS	ASPECTS	Infarct volume(ml)	90day mRS	Hemorrhage
3	7	19	1	None

Right side

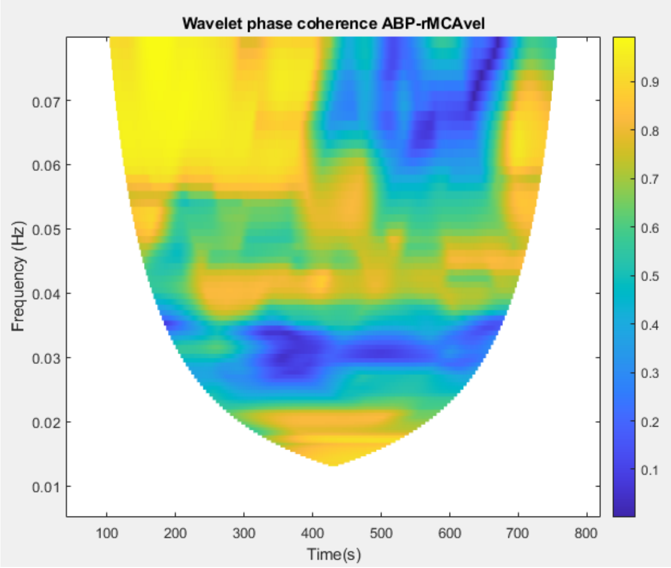

Left side

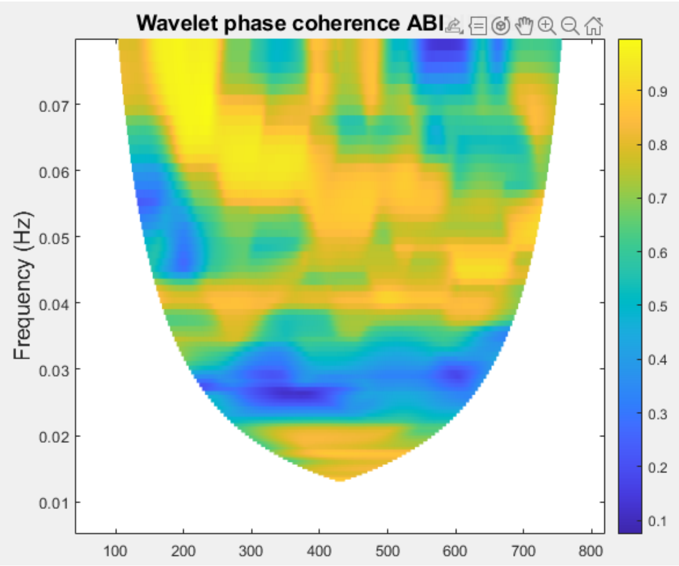

Age	Sex	Occlusion	Ipsilateral Median SI	Ipsilateral Peak SI	Contralateral Median SI	Contralateral Peak SI
56	Male	Right M1	0.40	0.90	0.36	0.75

24h NIHSS	ASPECTS	Infarct volume(ml)	90day mRS	Hemorrhage
3	7	24	0	HI1

Right side

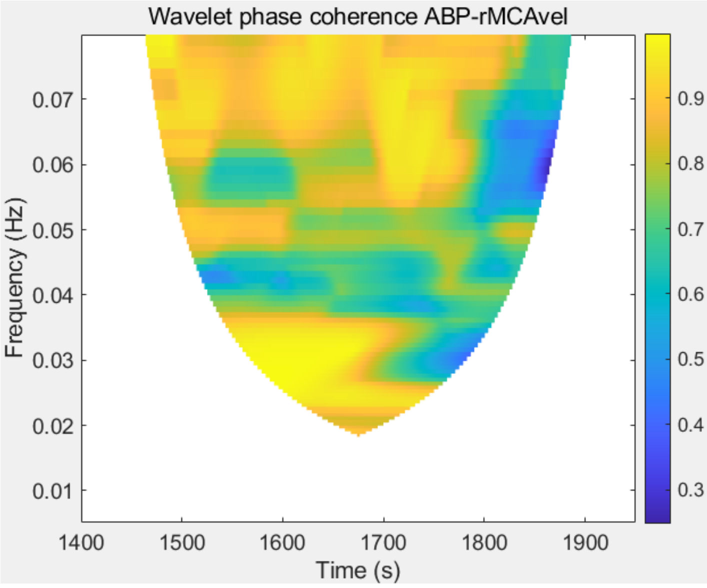

Left side

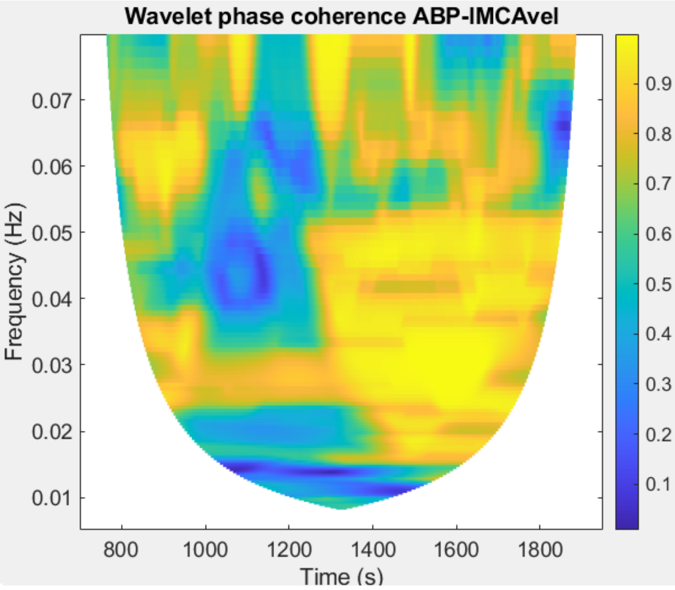

Age	Sex	Occlusion	Ipsilateral Median SI	Ipsilateral Peak SI	Contralateral Median SI	Contralateral Peak SI
65	Male	Left ICA+M1	0.22	0.63	0.20	0.64

24h NIHSS	ASPECTS	Infarct volume(ml)	90day mRS	Hemorrhage
5	7	17	2	PH1

Right side

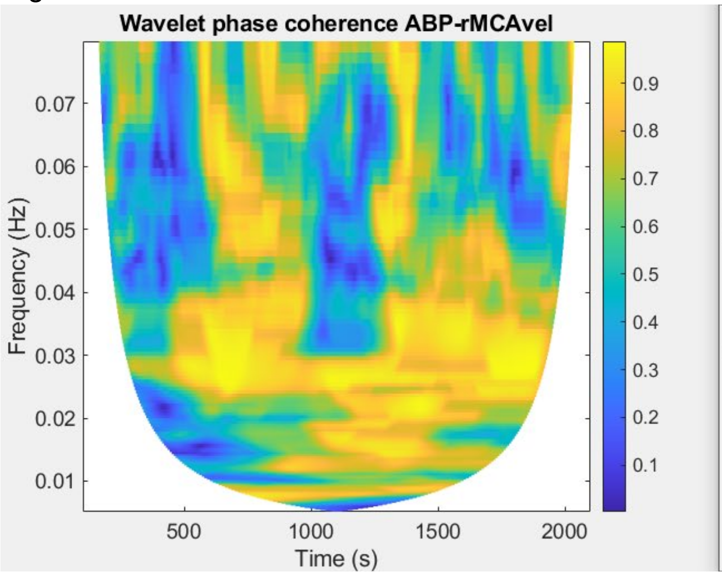

Left side

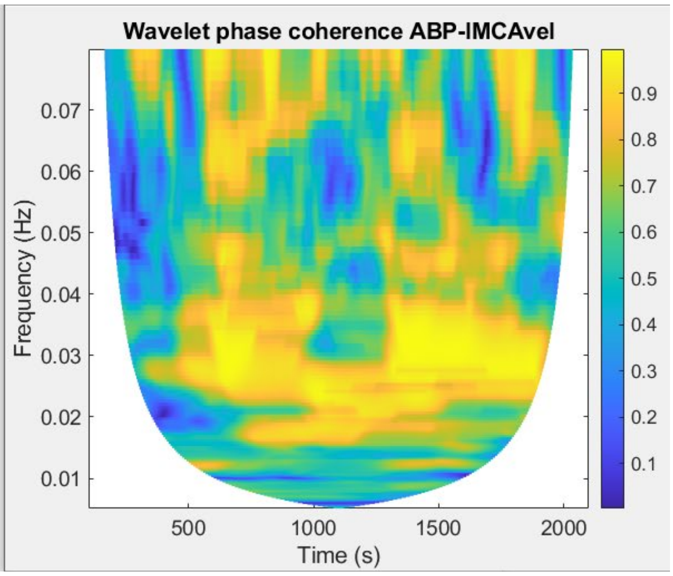

Age	Sex	Occlusion	Ipsilateral Median SI	Ipsilateral Peak SI	Contralateral Median SI	Contralateral Peak SI
71	Male	Left M1	0.72	0.93	0.60	0.92

24h NIHSS	ASPECTS	Infarct volume(ml)	90day mRS	Hemorrhage
16	4	46	4	PH1

Right side

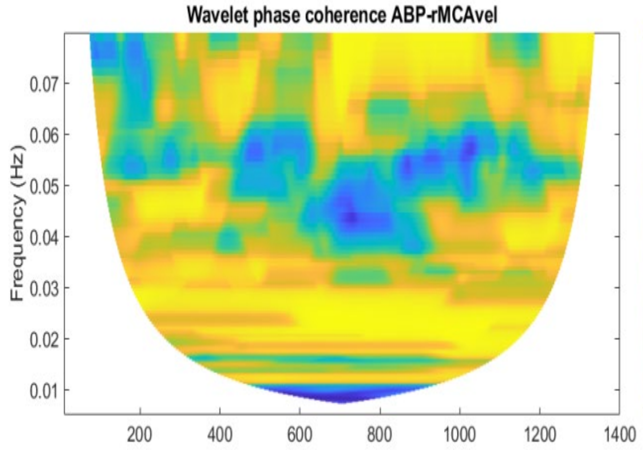

Left side

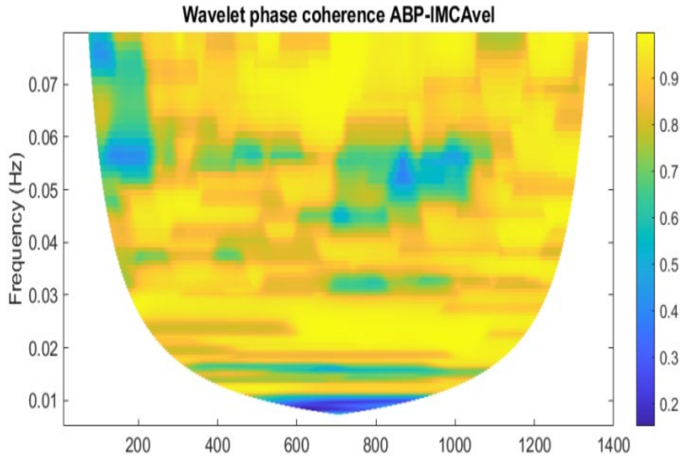

Age	Sex	Occlusion	Ipsilateral Median SI	Ipsilateral Peak SI	Contralateral Median SI	Contralateral Peak SI
76	Male	Left M1	0.54	0.80	0.13	0.30

24h NIHSS	ASPECTS	Infarct volume(ml)	90day mRS	Hemorrhage
22	3	96	4	PH1

Right side

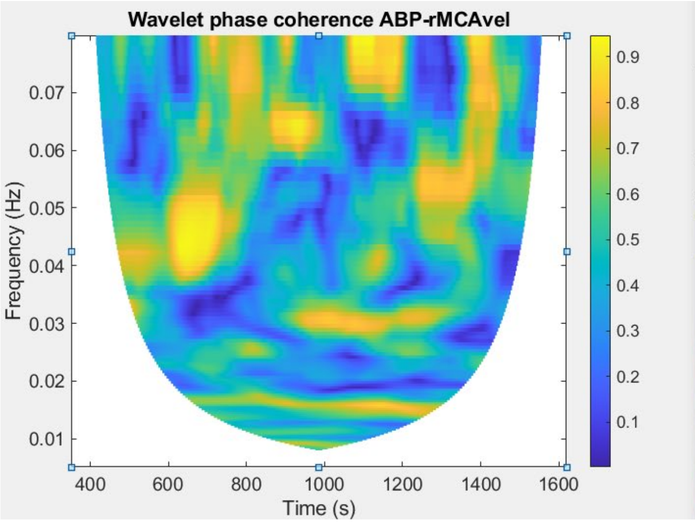

Left side

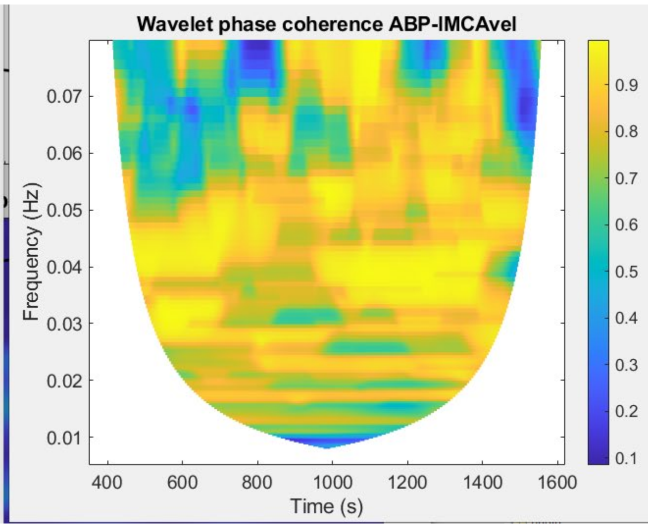

Age	Sex	Occlusion	Ipsilateral Median SI	Ipsilateral Peak SI	Contralateral Median SI	Contralateral Peak SI
76	Male	Right tICA	0.59	0.90	0.25	0.47

24h NIHSS	ASPECTS	Infarct volume(ml)	90day mRS	Hemorrhage
24	0		6	None

Right side

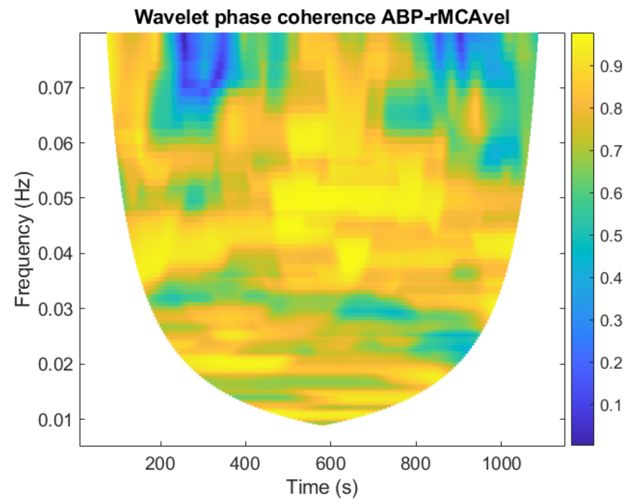

Left side

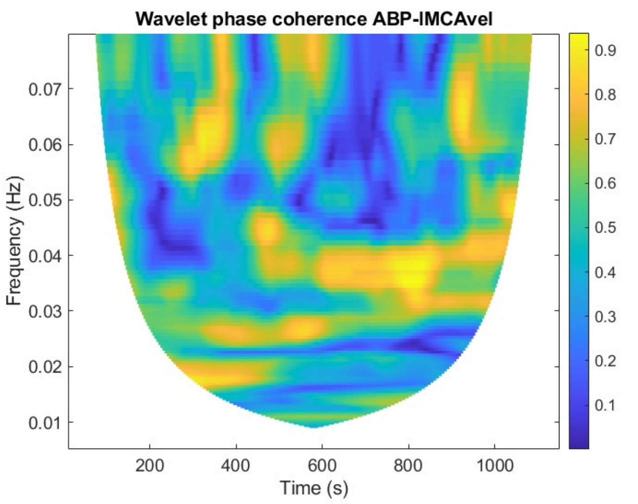

Age	Sex	Occlusion	Ipsilateral Median SI	Ipsilateral Peak SI	Contralateral Median SI	Contralateral Peak SI
72	Female	Left M1	0.54	0.86	0.54	0.82

24h NIHSS	ASPECTS	Infarct volume(ml)	90day mRS	Hemorrhage
16	4	51	2	None

Right side

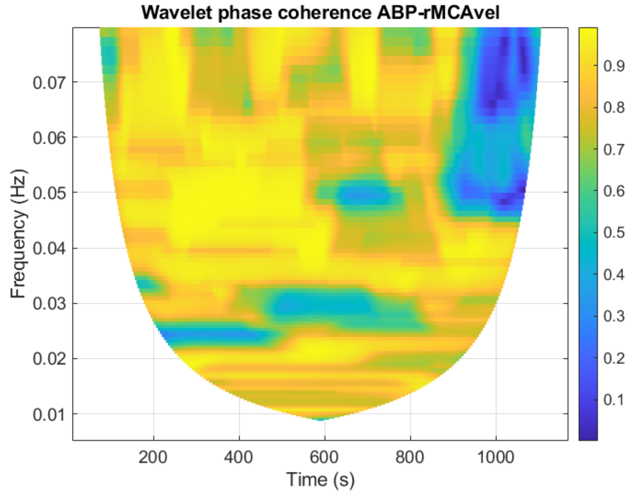

Left side

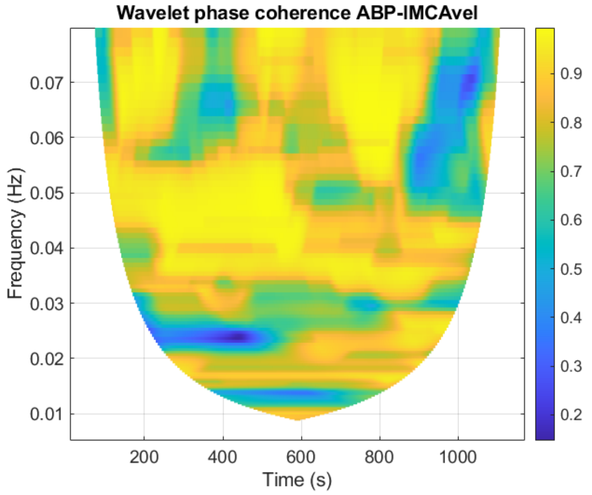

Age	Sex	Occlusion	Ipsilateral Median SI	Ipsilateral Peak SI	Contralateral Median SI	Contralateral Peak SI
76	Male	Left M1	0.65	0.93	0.58	0.91

24h NIHSS	ASPECTS	Infarct volume(ml)	90day mRS	Hemorrhage
28	4		6	SAH

Right side

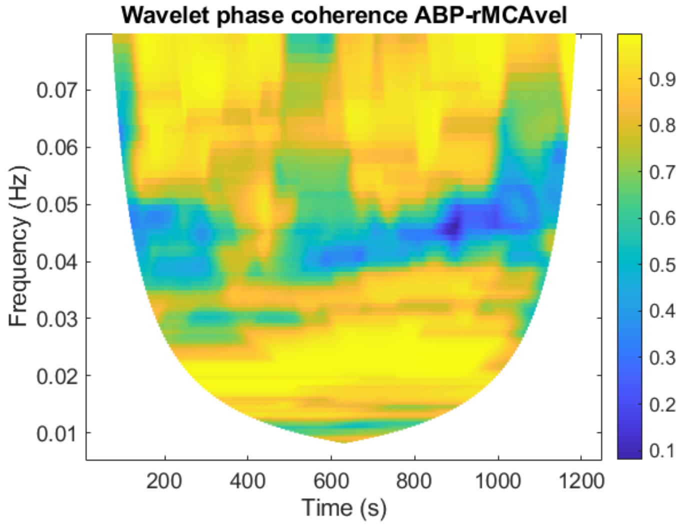

Left side

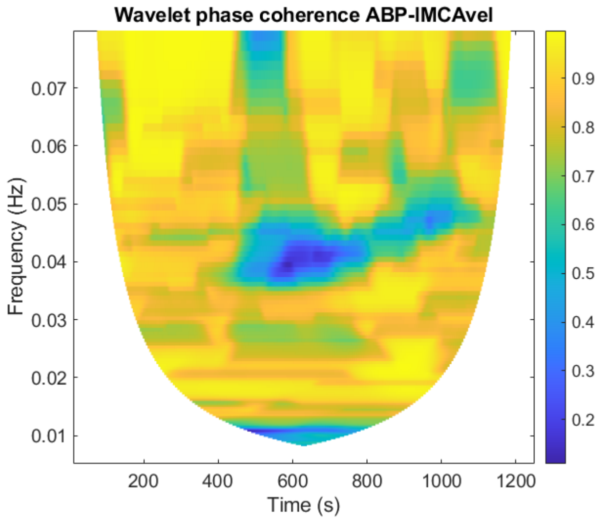

Age	Sex	Occlusion	Ipsilateral Median SI	Ipsilateral Peak SI	Contralateral Median SI	Contralateral Peak SI
72	Female	Right M1	0.10	0.69	0.17	0.63

24h NIHSS	ASPECTS	Infarct volume(ml)	90day mRS	Hemorrhage
0	10		2	None

Right side

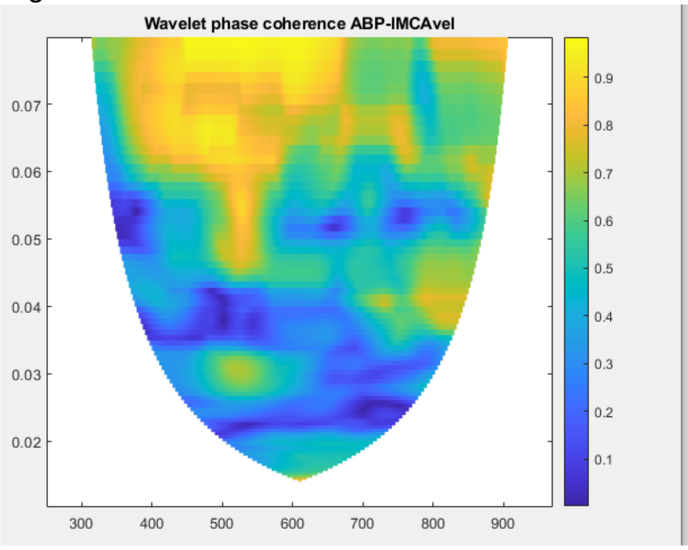

Left side

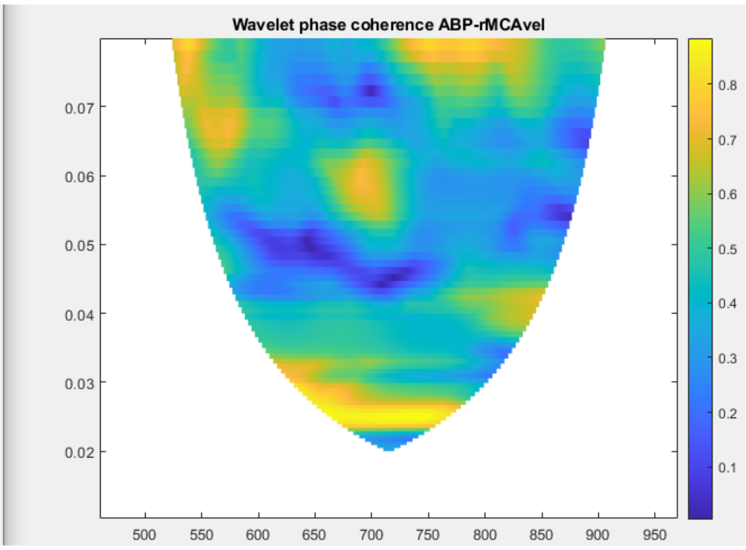

Age	Sex	Occlusion	Ipsilateral Median SI	Ipsilateral Peak SI	Contralateral Median SI	Contralateral Peak SI
59	Female	Right M1	0.12	0.40	0.13	0.52

24h NIHSS	ASPECTS	Infarct volume(ml)	90day mRS	Hemorrhage
0	8	8	0	None

Right side

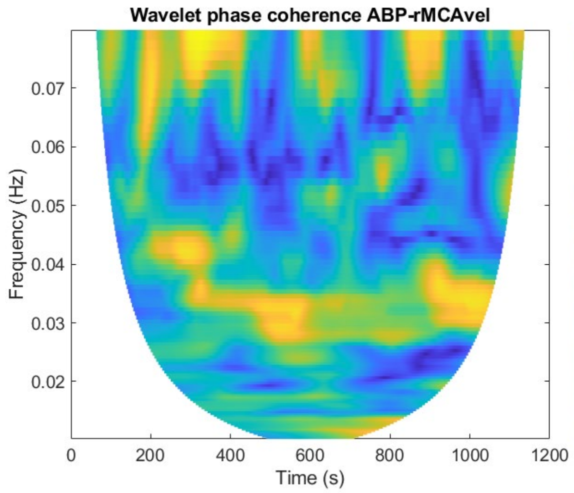

Left side

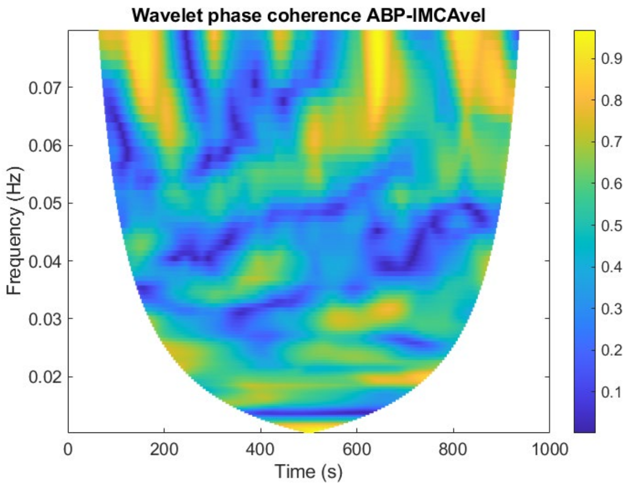

Age	Sex	Occlusion	Ipsilateral Median SI	Ipsilateral Peak SI	Contralateral Median SI	Contralateral Peak SI
80	Male	Right M1	0.90	1	0.68	0.99

24h NIHSS	ASPECTS	Infarct volume(ml)	90day mRS	Hemorrhage
17	3	90	6	PH1

Right side

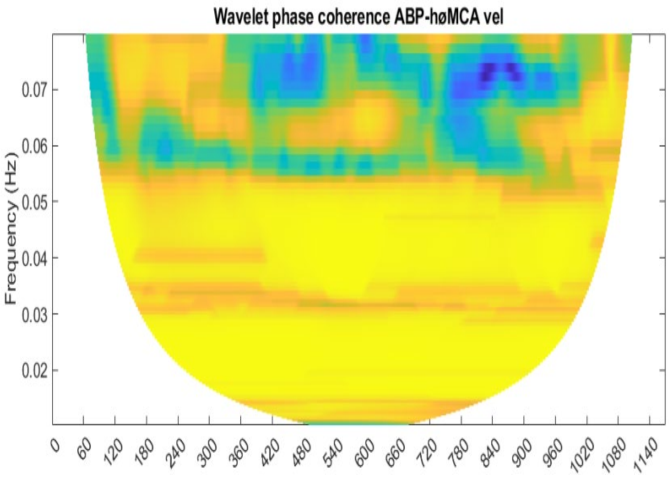

Left side

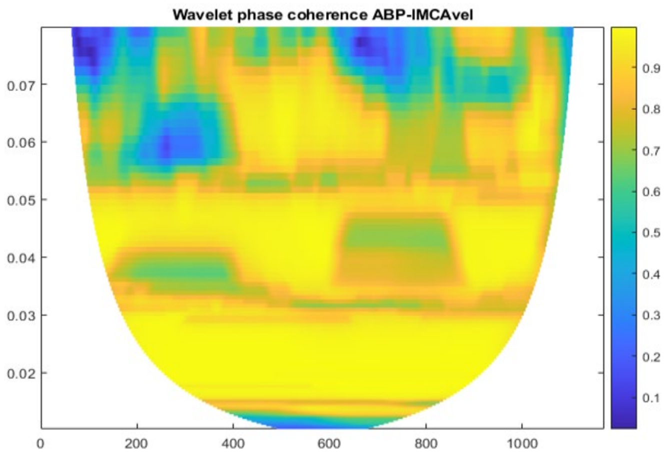

Age	Sex	Occlusion	Ipsilateral Median SI	Ipsilateral Peak SI	Contralateral Median SI	Contralateral Peak SI
62	Male	Left tICA	0.47	0.76	0.30	0.77

24h NIHSS	ASPECTS	Infarct volume(ml)	90day mRS	Hemorrhage
22	1	209	6	HI1

Right side

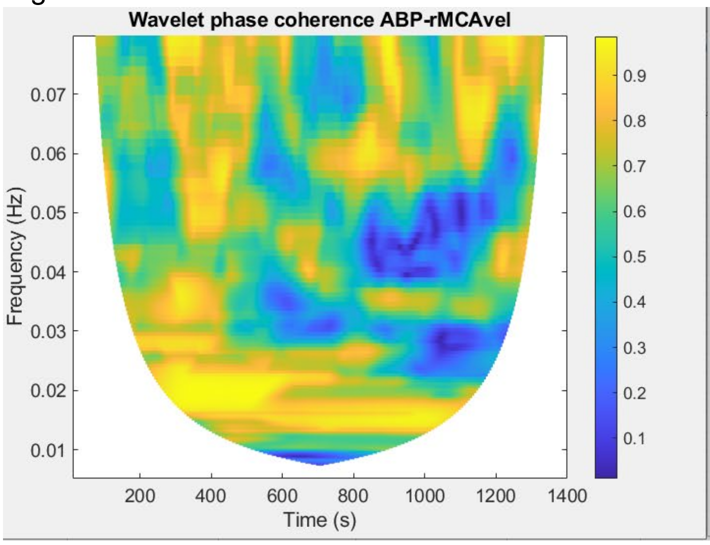

Left side

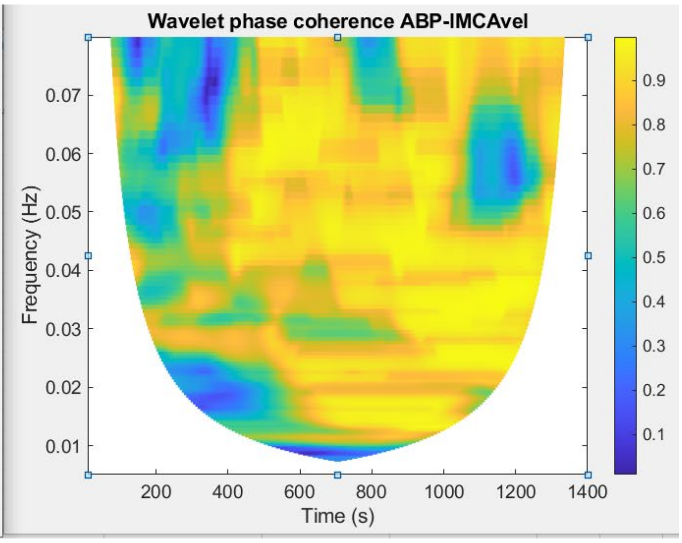

Age	Sex	Occlusion	Ipsilateral Median SI	Ipsilateral Peak SI	Contralateral Median SI	Contralateral Peak SI
85	Female	Left M1	0.75	0.95	0.64	0.96

24h NIHSS	ASPECTS	Infarct volume(ml)	90day mRS	Hemorrhage
9	6	21	2	HI2

Right side

Left side

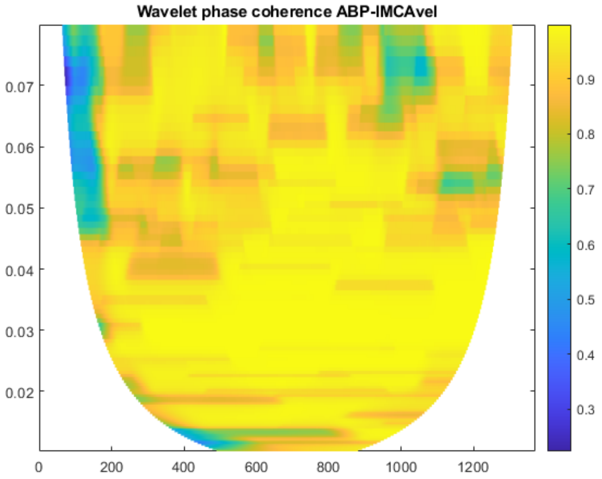

Age	Sex	Occlusion	Ipsilateral Median SI	Ipsilateral Peak SI	Contralateral Median SI	Contralateral Peak SI
58	Male	Right tICA	0.67	0.90	0.72	0.86

24h NIHSS	ASPECTS	Infarct volume(ml)	90day mRS	Hemorrhage
0	9	2	0	None

Right side

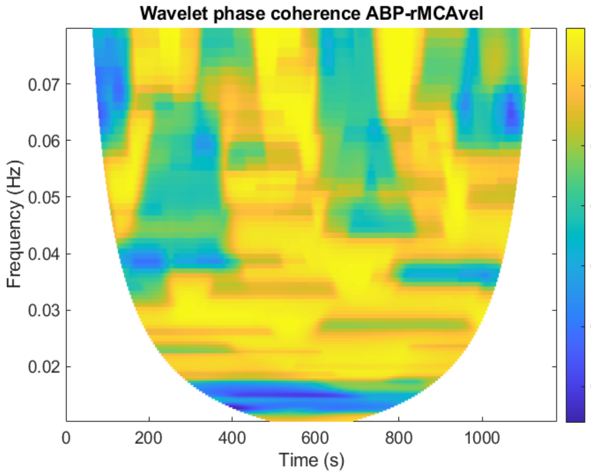

Left side

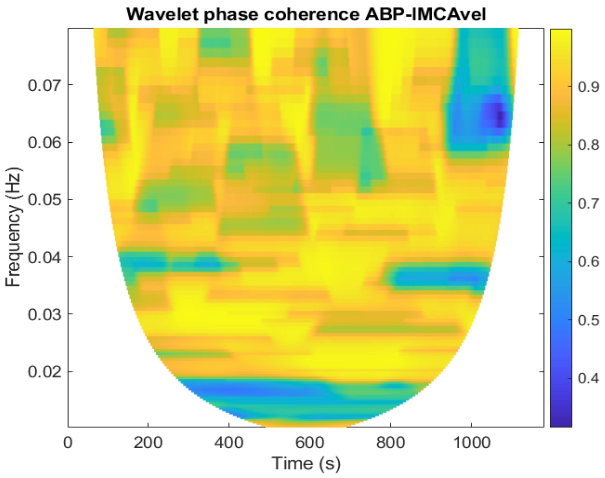

Age	Sex	Occlusion	Ipsilateral Median SI	Ipsilateral Peak SI	Contralateral Median SI	Contralateral Peak SI
75	Male	Left M2	0.53	0.88	0.47	0.85

24h NIHSS	ASPECTS	Infarct volume(ml)	90day mRS	Hemorrhage
11	6		3	SAH

Right side

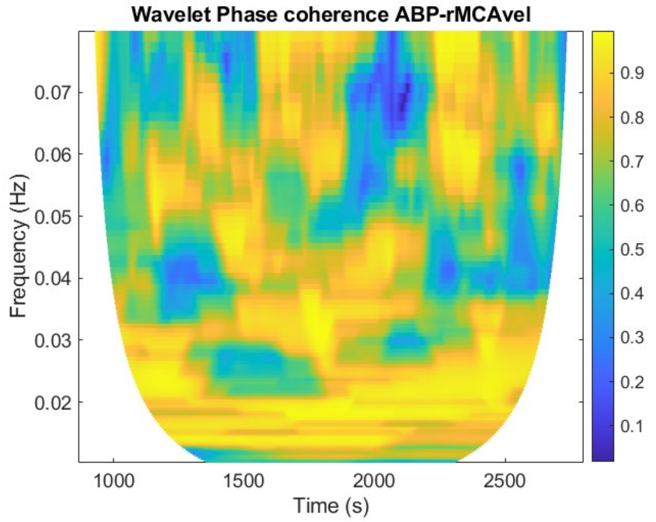

Left side

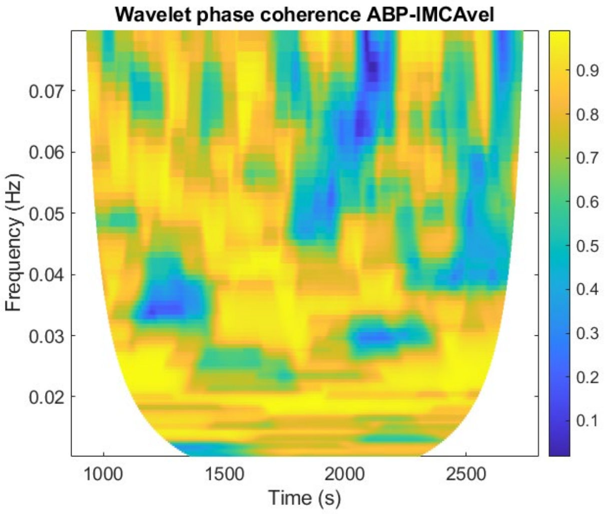

Age	Sex	Occlusion	Ipsilateral Median SI	Ipsilateral Peak SI	Contralateral Median SI	Contralateral Peak SI
55	Male	Right M2	0.32	0.67	0.22	0.59

24h NIHSS	ASPECTS	Infarct volume(ml)	90day mRS	Hemorrhage
3	6	71	2	HI1

Right side

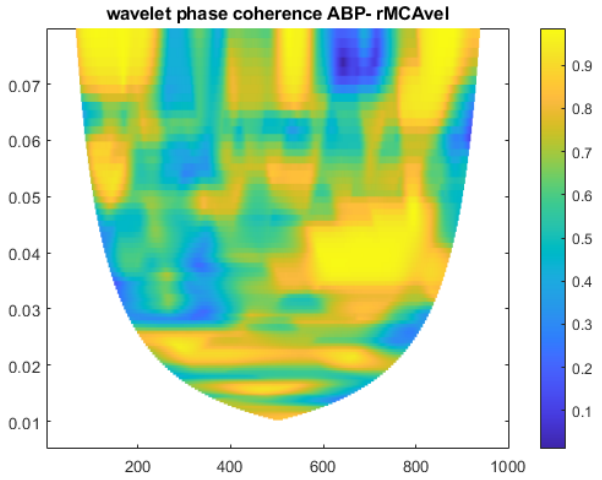

Left side

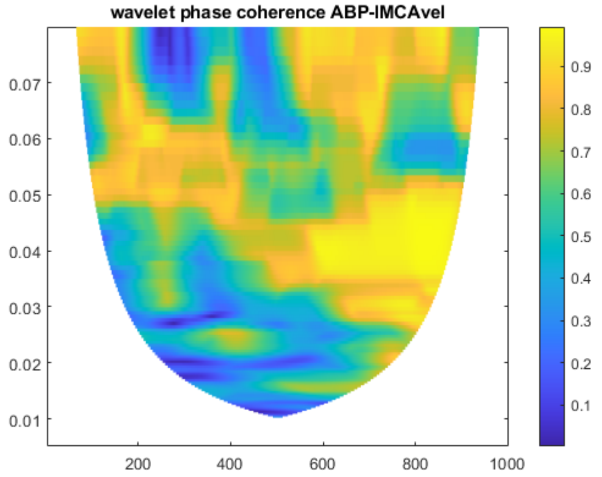

Age	Sex	Occlusion	Ipsilateral Median SI	Ipsilateral Peak SI	Contralateral Median SI	Contralateral Peak SI
67	Male	Left M2	0.57	0.73	0.34	0.81

24h NIHSS	ASPECTS	Infarct volume(ml)	90day mRS	Hemorrhage
0	10	0	0	None

Right side

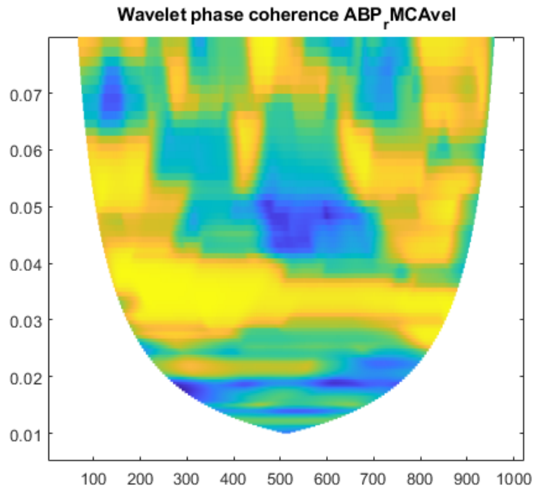

Left side

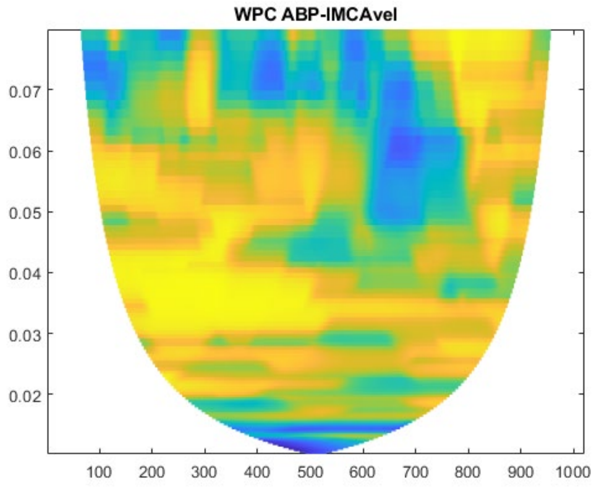

Age	Sex	Occlusion	Ipsilateral Median SI	Ipsilateral Peak SI	Contralateral Median SI	Contralateral Peak SI
77	Female	Right M1	0.45	0.70	0.41	0.77

24h NIHSS	ASPECTS	Infarct volume(ml)	90day mRS	Hemorrhage
6	7	26	2	HI1

Right side

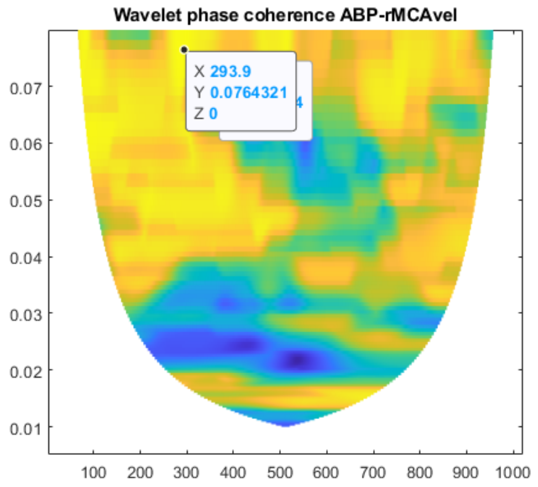

Left side

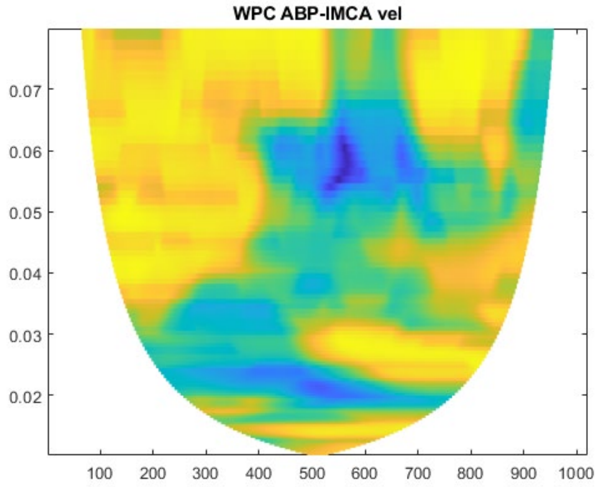

Age	Sex	Occlusion	Ipsilateral Median SI	Ipsilateral Peak SI	Contralateral Median SI	Contralateral Peak SI
57	Female	Right M1	0.20	0.55		

24h NIHSS	ASPECTS	Infarct volume(ml)	90day mRS	Hemorrhage
2	9	6		None

Right side

Left side

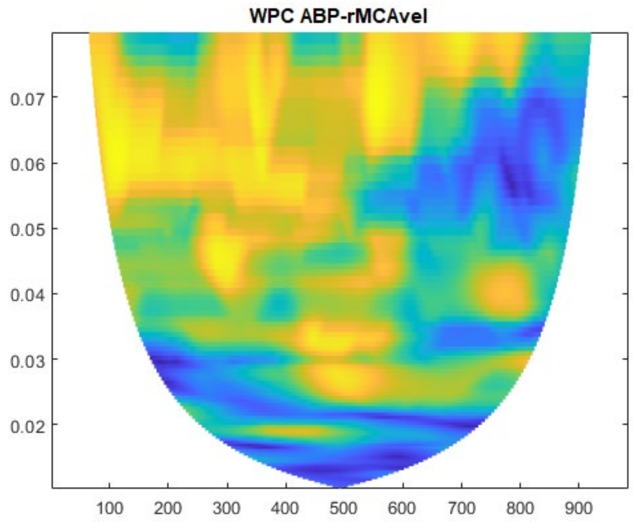

Age	Sex	Occlusion	Ipsilateral Median SI	Ipsilateral Peak SI	Contralateral Median SI	Contralateral Peak SI
58	Female	Left M1	0.36	0.78	0.35	0.56

24h NIHSS	ASPECTS	Infarct volume(ml)	90day mRS	Hemorrhage
1	6	11	0	HI2

Right side

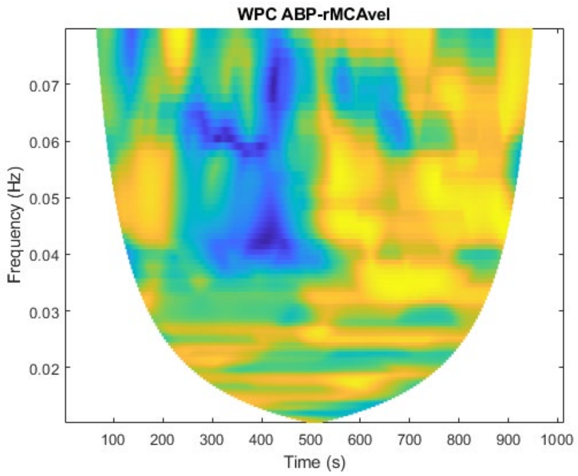

Left side

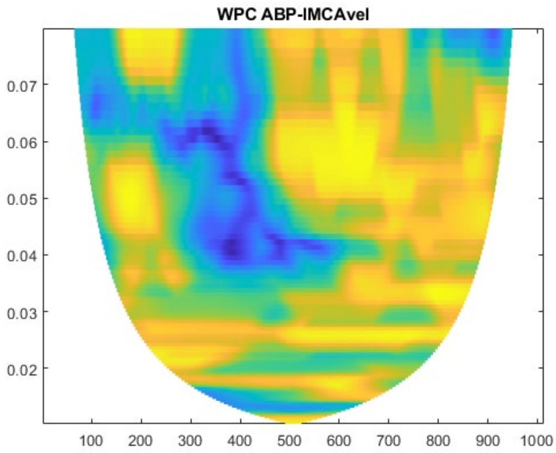

Age	Sex	Occlusion	Ipsilateral Median SI	Ipsilateral Peak SI	Contralateral Median SI	Contralateral Peak SI
77	Male	Left M1	0.46	0.73	0.63	0.91

24h NIHSS	ASPECTS	Infarct volume(ml)	90day mRS	Hemorrhage
8	5	76	3	HI2

Right side

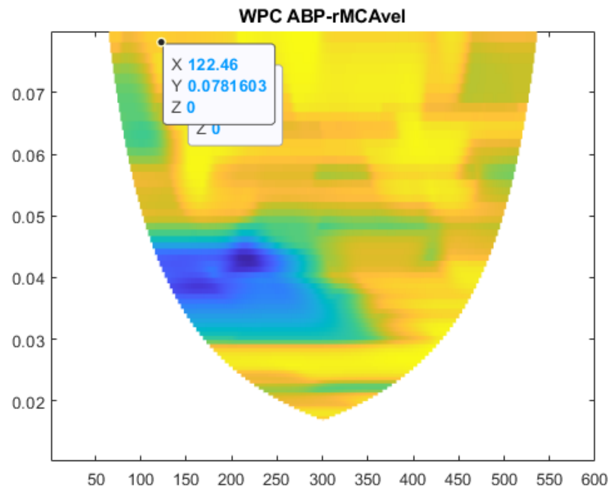

Left side

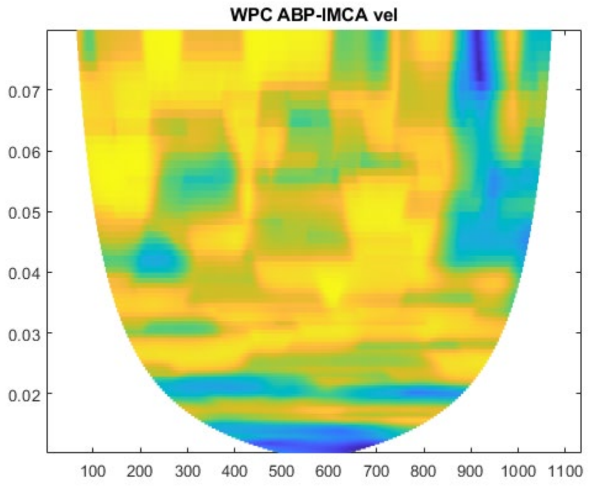

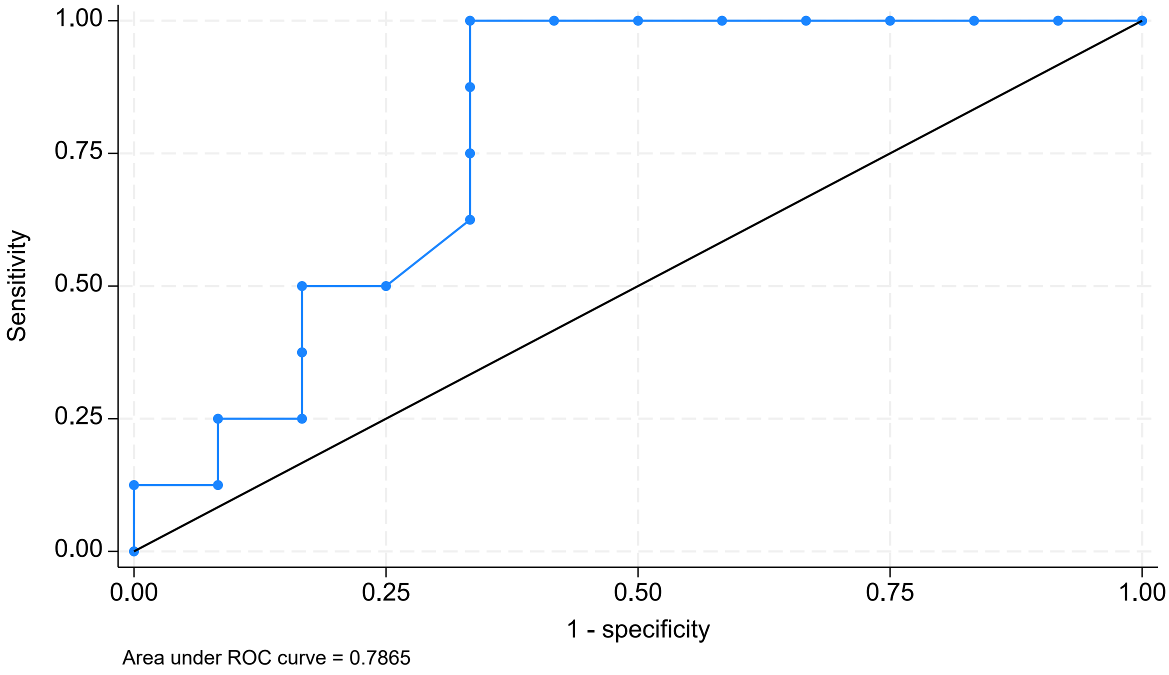

Figure S1 Receiver operating characteristic (ROC) curve illustrating the performance of SI in predicting unfavorable 90-day mRS.
